# Beyond monotherapy: multimodal strategies integrating immune checkpoint inhibitors in lymphoma management

**DOI:** 10.3389/fimmu.2025.1713199

**Published:** 2025-11-20

**Authors:** Yuxiao Li, Ruixin Zheng, Bing Pan, Xiaobo Wang, Lina Zhang, Haiying Gao, Li Li

**Affiliations:** 1 Department of Hematology, The Second Hospital of Dalian Medical University, Dalian, China; 2 Department of General Medicine, The Second Hospital of Dalian Medical University, Dalian, China

**Keywords:** immune checkpoint inhibitors, lymphoma, combination therapy, PD-1/PD-L1, CTLA-4

## Abstract

The advent of immune checkpoint inhibitors (ICIs) has revolutionized lymphoma therapy, though efficacy varies markedly between Hodgkin lymphoma (HL) and non-Hodgkin lymphoma (NHL). ICIs targeting the programmed cell death protein 1/programmed death-ligand 1 (PD-1/PD-L1) pathway show significant efficacy in HL, but limited benefit in NHL subtypes including diffuse large B-cell lymphoma; in T-cell lymphomas and natural killer (NK) cell lymphomas, PD-1 inhibitors demonstrate significant efficacy in extranodal NK/T-cell lymphoma, but response rates remain limited for most peripheral T-cell lymphoma subtypes. The PD-1/PD-L1 axis is central to lymphoma immunotherapy: PD-1/PD-L1 blockade counters tumor immune evasion, whereas CTLA-4 inhibition enhances early T-cell activation in lymphoid tissues. Additional checkpoints also contribute to disease progression by mediating T-cell exhaustion, underscoring their therapeutic relevance. This review delineates the mechanistic rationale and clinical implications of combining ICIs with conventional therapies (chemotherapy, radiotherapy), targeted agents, and emerging modalities. Synergistic combinations have shown promise in overcoming resistance and amplifying antitumor immunity. Clinical trials highlight PD-1 inhibitor-chemotherapy/radiotherapy regimens improving response and survival rates in select lymphomas. Immuno-combination therapies achieve superior efficacy in specific subtypes despite heightened immune-related adverse events. By synthesizing evidence across different combination approaches, this perspective provides clinicians with an integrative framework that transcends traditional disease subtype boundaries, offering broader insights for therapeutic decision-making in lymphoma immunotherapy. Current challenges include developing predictive biomarkers and optimizing management of immune-related adverse events. Collectively, integrating ICIs with complementary modalities offers transformative potential, yet requires rigorous mechanistic exploration and clinical validation to maximize therapeutic index and durability of responses.

## Introduction

Hodgkin lymphoma (HL) and non-Hodgkin lymphoma (NHL) are examples of lymphomas, which are malignant tumors that start in the lymphatic system. Even while traditional chemotherapy and radiation therapy have been successful in treating lymphoma, a sizable percentage of patients remain insensitive to or eventually develop resistance to these treatments. As a result, research is now focused on investigating novel therapeutic approaches to enhance patient survival quality and efficacy.

The area of tumor therapy has seen a radical transformation in recent decades, with the advent of a new age of tumor immunotherapy signaled by the development and use of Immune Checkpoint Inhibitors (ICIs). Because of its distinct immunological milieu and immune escape mechanism, lymphoma has emerged as one of the most active tumor types for immune checkpoint inhibitor development.

**Figure 1:**
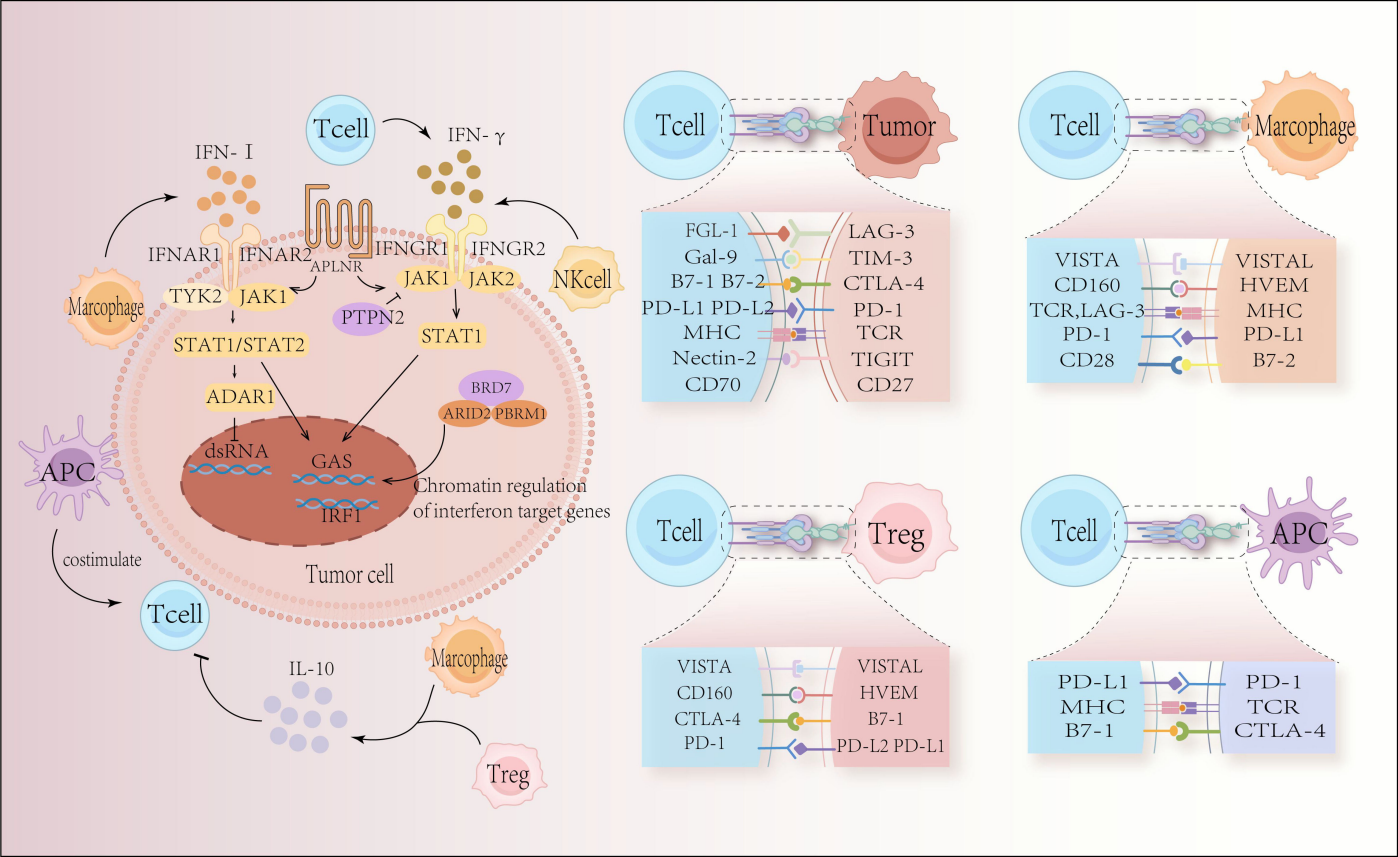
Tumor-intrinsic escape mechanisms and the interaction between immune cells and tumor cells through immune checkpoints. In response to immune checkpoint blockage and T-cell proliferation-based immunotherapy, tumor-endogenous interferon signaling plays a crucial role. The success or failure of immune checkpoint blockade depends on elements of the type I interferon signaling pathways and interferon gamma (IFNγ) signaling pathways, including Janus kinase 1 (JAK 1), JAK 2, signal transducer and activator of transcription 1 (STAT 1), and IFNγ receptor I (IFNGR 1) and IFNGR 2. The surface receptor apelin receptor (APLNR) regulates upstream sensitivity to IFNγ and type I interferon signaling; the tyrosine protein phosphatase non-receptor type 2 (PTPN2) regulates upstream sensitivity to IFNγ signaling; the bromodomain-containing protein 7 (BRD7) and DNA-binding subunits, AT-rich interaction domain 2 (ARID2) and Polybromo-1 (PBRM1), are part of the chromatin remodeling complex polybromo-associated BAF (PBAF) and are involved in the regulation of IFNγ target gene regulation; double-stranded RNA (dsRNA)-specific adenosine deaminase (ADAR1), negatively regulates endogenous dsRNA levels. Type I interferons and IFNγ signaling converge at the IFNγ activation site (GAS) in DNA, activating transcriptional regulators including interferon regulatory factor 1 (IRF 1). This leads to critical interferon signaling outputs. The immunological component of the tumor microenvironment is comprised of many types of immune cells that are strongly associated with anti-tumor immunity. Tumor cell expression of immune checkpoint proteins disrupts anti-tumor immunity, reduces T cell immunological function, and promotes cancer cell proliferation and expansion.

Immune checkpoint inhibitors have shown previously remarkable clinical success in the treatment of lymphoma. Specifically, these drugs target programmed cell death protein 1 (PD-1) and its ligand (PD-Ligand 1, PD-L1). These medications increase T cell cytotoxicity by inhibiting the PD-1/PD-L1 pathway, which causes tumor cell lysis (1). Tumor endogenous interferon signaling plays a crucial role in response to immune checkpoint blockade and T cell proliferation-based immunotherapy (**Figure 1**). The significant role that PD-1 and PD-L1 play in immunosuppression has been confirmed in recent years by the impressive effectiveness of PD-1 or PD-L1 blockade immunotherapy in treating many kinds of tumors, including hematologic malignancies (2, 3).

However, monotherapy has a low response rate, and some patients develop medication resistance. Combination therapy tactics have been developed to improve the immune system ability to fight tumors by combining multiple drugs and inhibiting tumor immune escape from diverse directions. To overcome the limitations of monotherapy, researchers have proposed multiple combination treatment regimens. This combination therapy comprises new immunotherapeutic medications in addition to chemotherapy and radiation. Particularly for lymphoma, studies have demonstrated that combination therapy may yield superior efficacy compared to monotherapy.

This review briefly introduces immune checkpoint molecules and the mechanisms of action of their inhibitors. And it thoroughly examines the synergistic antitumor mechanisms and clinical research outcomes when immune checkpoint inhibitors are combined with other therapeutic approaches in lymphoma treatment. This paper aims to transcend the mere enumeration of individual research findings. Through a comparative and critical analysis of existing evidence on combination therapy models, it seeks to derive insights from both successful and unsuccessful clinical trials. This approach provides clinicians with a framework for selecting personalized combination treatments, ultimately aiming to deliver more effective therapeutic options for lymphoma patients.

## Immunization checkpoints and their functions

### CTLA-4

Cytotoxic T lymphocyte-associated antigen 4 (CTLA-4), the first immune checkpoint receptor to be clinically targeted, is a transmembrane receptor predominantly expressed on activated T lymphocytes and regulatory T cells (Tregs) (**Figure 1**). The expression and function of CTLA-4 are closely associated with T-cell activation. T-cell activation is initiated when the T cell receptor (TCR) recognizes an antigen presented by the major histocompatibility complex (MHC) on the antigen-presenting cell (APC), leading to the binding of CD28 on the T cell to B7 on the APC. This sets off a signaling cascade reaction (4). CTLA-4 in the intracellular compartment is promptly transferred to the immunological synapse as the TCR activates T-cells, and the greater the TCR signal, the more CTLA-4 is transferred to the immune synapse (5).

Once at the synapse, CTLA-4 stabilizes by binding to CD80 and CD86 ligands, thereby accumulating and effectively competing with CD28 (6). CD28 or CTLA-4 is predominantly targeted to the immunizing synapse as a result of variations in ligand binding affinity. While CD86 is the predominant ligand for CD28 localization, CD80 is the primary ligand for CTLA-4 localization in synapses (6). By reducing CD28 positive co-stimulation, CTLA-4 restricts CD28 downstream signaling, which is mostly mediated by PI3K and AKT (5). This competitive suppression of CD28 by CTLA-4 is crucial for modulating the immune response, as CTLA-4 has a higher affinity for CD80/CD86 than CD28 (7). This mechanism is particularly important during the initiation and progression of infections and autoimmune diseases, where the balance of T-cell immunity is maintained by the synergistic interaction between CTLA-4 and CD28 (4).

CTLA-4 regulates T cell activation both extracellularly and through intrinsic mechanisms, primarily involving regulatory T cells (Tregs) (8). CTLA-4 is a crucial functional molecule in Treg-mediated immunological tolerance, as evidenced by the fact that Treg-specific deletion of CTLA-4 causes aberrant T cell activation and autoimmunity (9, 10). Additionally, CTLA-4 can rapidly extract CD80/CD86 from APCs through trans-endocytosis, further contributing to immune regulation (11). In the context of cancer immunotherapy, blocking CTLA-4 enhances the proliferation and activation of T lymphocytes targeting tumor cells (12).

Solution Nuclear Magnetic Resonance (NMR) spectroscopy was used to establish the initial structure of CTLA-4, which revealed an Ig-like V (variable) type structural domain with two disulfide bond connecting the two β-folds of the V-fold 5. Later, a different CTLA-4 carrier shape in the physiological dimerization stage was reported (13). Notably, while the CD80-binding conformation of CTLA-4 is highly similar to its apo form, binding to CD86 requires significant structural changes, particularly in the FG loop (13–15). Recent reports have also identified several CTLA-4 structures that bind to monoclonal antibodies (PDB id: 5GGV, 5TRU, 5XJ3, and 6RP8) (16–18). According to these structures, CD80 and CD86 are directly competed with by CTLA-4 inhibitors at their binding surfaces, which sterically displaces them and inhibits their ability to engage with CTLA-4 5.

In recent years, CTLA-4 has emerged as a crucial target in immune checkpoint-based therapies, particularly in cancer treatment, through the use of monoclonal antibodies targeting the CTLA-4 molecule. Moreover, the management of autoimmune diseases is closely linked to this regulatory molecule (19). Elucidating the immunoregulatory mechanisms and functions of CTLA-4 in these conditions will facilitate the identification of effective immunotherapeutic targets for autoimmune diseases.

### PD-1/PD-L1

PD-1 is one of the most talked about immune checkpoint molecules today. It was first identified in 1992 by T. Honjo and colleagues at Kyoto University as an apoptosis-associated gene (20). PD-1 is expressed as a monomer on T cells, where it inhibits the synthesis of interleukin-2 (IL-2) and T-cell proliferation 3. Structurally, PD-1 comprises one immunoglobulin (Ig) V-like domain, a transmembrane domain, and an intracellular domain3.

PD-1 interacts with two ligands: PD-L1 and PD-L2. PD-L1 is broadly expressed across various cell types, including non-hematopoietic cells such as vascular endothelium, pancreatic islet cells, placental syncytiotrophoblasts, and keratinocytes, as well as hematopoietic cells like T cells, B cells, dendritic cells, and macrophages (**Figure 1**). In normal tissues, PD-L1 expression is crucial for maintaining physiological peripheral immune tolerance, regulating tissue autoimmune responses, and preventing persistent inflammatory reactions following tissue damage. Tumor cells can also exploit PD-L1 as a molecular “shield” to evade immune surveillance and reduce T-cell-mediated cytotoxicity. In contrast, PD-L2 has garnered less attention in cancer immunotherapy due to its low constitutive expression and more restricted expression pattern, primarily in dendritic cells, macrophages, and B cells (2, 21).

PD-1 is an essential checkpoint that regulates the T-cell-mediated anti-tumor immune response in the tumor microenvironment. Through TCR downstream signaling, it is expressed on the surface of activated T cells. After attaching itself to the ligand PD-L1, it sends out inhibitory signals to regulate T-cell activation. When PD-1 extracellular structural domains interact with PD-L1, PD-1 undergoes a conformational shift that phosphorylates cytoplasmic Immunoreceptor Tyrosine-based Inhibitory Motif (ITIM) and Immunoreceptor Tyrosine-based Switch Motif (ITSM). Recruitment of protein tyrosine phosphatase 2 (SHP-2) and SHP-1, which include the Src homology region 2. Following recruitment, SHP-1/2 dephosphorylates ζ-associated protein 70 (ZAP70), a downstream participant in the TCR signaling cascade that suppresses the RAS/MEK/Erk, PI3K/Akt, and protein kinase C-θ (PKC-θ) pathways. Ultimately, decreased T-cell activity is tightly linked to the PD-1-mediated inhibitory pathway (22–24).

PD-L1 and PD-L2 are frequently overexpressed by malignant lymphoma cells to protect against activated intratumoral T cells. We summarized the immune checkpoint expression profiles of different lymphocyte subtypes, as shown in **Table 1**. Monoclonal antibodies targeting PD-1 can remove the inhibitory signals sent to T cells, thereby reactivating intratumoral T cells that target malignant clones. However, the therapeutic benefit of PD-1 blockade varies among different lymphoma subtypes. For instance, HL has shown durable responses to anti-PD-1 antibodies (31–33), whereas other lymphoma subtypes, such as small lymphocytic lymphoma (SLL), peripheral T-cell lymphoma (PTCL), follicular lymphoma (FL), and diffuse large B-cell lymphoma (DLBCL), have demonstrated limited therapeutic efficacy from PD-1 blockade (34).

**Table 1:** Immune checkpoint landscapes and clinical implications across lymphoma subtypes.

Lymphoma subtype	Primary checkpoint expression	Other key checkpoint expression	Cellular sources in the TME	Clinical implications and future directions
Classical Hodgkin Lymphoma (cHL) (25)	PD-L1: Very High PD-L2: Very High PD-1: High (on T cells)	TIGIT: High TIM-3: High LAG-3: High CTLA-4: High	•H/RS cells •Tumor-associated macrophages •Exhausted T cells	•Paradigm for successful PD-1 blockade. •Co-expression of multiple inhibitory receptors provides a strong rationale for combination immunotherapies (e.g., PD-1 + TIGIT inhibitors).
Primary Mediastinal Large B-Cell Lymphoma (PMBCL) (26)	PD-L1: Very High PD-L2: High	LAG-3: High TIGIT: Moderate/High	•Tumor B cells	•High response rates to PD-1 inhibitors, enabling chemotherapy-free strategies. •Targeting LAG-3 or TIGIT may help overcome resistance.
Diffuse Large B-Cell Lymphoma (DLBCL) (27)	PD-L1: Variable (Higher in non-GCB, EBV+ cases) PD-1: High (on TILs)	TIM-3: High TIGIT: High CTLA-4: Variable	•Tumor B cells (subset) •TILs and myeloid cells	•Response heterogeneity necessitates biomarker development. •Combinations with TIM-3 or TIGIT inhibitors are under investigation to reverse T-cell exhaustion.
Follicular Lymphoma (FL) (28)	PD-1: Very High PD-L1: Low/Moderate	LAG-3: High TIGIT: High CTLA-4: High	•Follicular helper T cells (Tfh) •Exhausted T cells	•Modest efficacy of PD-1 monotherapy suggests compensatory pathways. •Novel strategies targeting ICOS/LAG-3 on Tfh cells or combining with CTLA-4 inhibition are emerging.
Mantle Cell Lymphoma (MCL) (29)	PD-L1: Generally Low PD-1: High (on T cells)	BTLA: High LAG-3: Moderate	•Tumor B cells •Non-malignant stromal cells	•Modest responses to PD-1 blockade monotherapy. •BTLA-HVEM interaction is a key immunosuppressive axis. Combining BTK inhibitors with ICIs is a major focus, despite challenges with toxicity.
Extranodal NK/T-cell Lymphoma (NKTCL) (30)	PD-L1: High	TIM-3: High LAG-3: High CTLA-4: Moderate/High	•Tumor NK/T cells •Infiltrating lymphocytes	•High response rates to PD-1 blockade. •EBV drives multiple checkpoints; PD-1 combined with TIM-3 or LAG-3 blockade is a promising strategy.

In most lymphoma patients, the only component leading to tumor immune evasion is not the PD-1/PD-L1 axis. Therefore, mere blocking PD-1/PD-L1 is insufficient to trigger a powerful immune response against the tumor. As a result, anti-PD-1 drugs are frequently utilized in combination with other therapeutic methods, including chemotherapy, radiation, and new ICIs.

### LAG-3

Following the clinical success of targeting CTLA-4 and PD-1, other co-inhibitory molecules have garnered increased attention, particularly lymphocyte activation gene-3 (LAG-3, CD223). LAG-3, first identified by Triebel and colleagues in 1990 (35), is a type I transmembrane protein composed of four immunoglobulin (Ig)-like structural domains (D1–D4). Notably, the D1 domain contains nine β-strands on the A, B, C, C′, C′, D, E, F, and G chains of the IgV fold. A distinctive feature of LAG-3 is the “extra loop,” a 30-amino acid sequence between the C and C’ chains, conserved in both human and mouse LAG-3, which has been implicated in the interaction with major histocompatibility complex class II (MHC II) (36, 37).

LAG-3 is not expressed on naïve T cells, but it can be induced on CD4+ and CD8+ T cells after antigen stimulation (35, 38). Its suppressive function is correlated with its expression level on the cell surface, highlighting the importance of regulating LAG-3 expression (39). Chronic infections with viruses, bacteria, and parasites cause continuous antigen exposure, which results in increased expression of LAG-3 and other inhibitory co-receptors on CD4+ and CD8+ T cells (40–43). These T cells, also known as depleted T cells, lose their tremendous effector function. Although less than PD-1 blockage, LAG-3 inhibition has been shown to activate depleting T cells and boost anti-infection immunity (42). Similarly, tumor-infiltrating T lymphocytes have high levels of several inhibitory co-receptors, including LAG-3, and are continuously exposed to tumor-associated antigens, which results in functional exhaustion (44, 45). The expression of LAG-3 on activated T cells is further upregulated by cytokines such as interleukin-2 (IL-2), IL-7, and IL-12 (46), and it is also observed in various suppressive CD4+ T cell subsets.

LAG-3 is primarily associated with the MHC-II molecule, which exhibits a greater affinity for LAG-3 compared to CD4 (36) (**Figure 1**). This interaction occurs through the D1 structural domain and negatively regulates T cell activation, cytotoxicity, and cytokine production (47). In cell adhesion experiments, a LAG-3-IG fusion protein has been shown to compete with this interaction (48). Although the specific signaling pathways remain unclear, LAG-3 binding to MHC-II transmits inhibitory signals that impede CD4 T cell activation (49). In addition to MHC-II, LAG-3 interacts with galactose lectin-3 (Gal-3), a C-type lectin that plays a critical role in regulating T-cell activation and is essential for suppressing CD8 T-cell cytotoxic function. Recently, fibrinogen-like protein 1 (FGL-1), released by the liver, has also been identified as another ligand for LAG-3 (50). Overall, LAG-3 inhibitory activity relies on its interactions with multiple ligands, suggesting that it primarily disrupts CD4: MHC-II connections.

The co-expression of LAG-3, PD-1, and TIM-3 is a determinant of T-cell exhaustion in NHL (51). Overexpression of LAG-3 is linked to worse clinical outcomes in follicular lymphoma. Yang et al. discovered that LAG-3+ T cells were nearly entirely derived from the PD-1+ population and that LAG-3 was expressed in a subgroup of intraneoplastic T cells in follicular lymphoma (44). IL-12 markedly increased the expression of LAG-3 on CD4+ or CD8+ T cells. Remarkably, CD8+ T cells within the tumor functioned better when the PD-1 and LAG-3 signaling pathways were blocked, which resulted in more interferon-γ (IFN-γ) and IL-2 production (52).

LAG-3 expression in distinct tumor-infiltrating lymphocyte (TIL) subpopulations, particularly in tumors with low MHC-II expression, may be critical for the development of a tolerogenic immune environment in classic Hodgkin lymphoma (cHL). Unfortunately, the high rate of response to checkpoint inhibition observed in cHL has not been replicated in patients with NHL, particularly in DLBCL. It is increasingly recognized that blocking LAG-3 alone may not be the ideal therapeutic strategy. The mechanism of synergy between anti-LAG-3 and anti-PD-1 therapy has attracted interest as a necessary condition for rationally designing anti-LAG-3 therapy with maximum efficacy and minimal side effects.

### TIM-3

In addition to the well-characterized immune checkpoint molecules, recent research has identified several other co-inhibitory molecules, including T-cell immunoglobulin and mucin domain-containing protein 3 (TIM-3). TIM-3 is a negative co-stimulatory molecule widely expressed across various immune cell types. Initially identified as a 60 kDa surface protein induced by IFN-γ on CD4+ and CD8+ T lymphocytes in mice (53), TIM-3 has since been found to be expressed on a diverse range of immune cells, including mast cells, monocytes/dendritic cells, regulatory T cells (Tregs), and natural killer (NK) cells (54–57) (**Figure 1**).

TIM-3 belongs to the TIM gene family, which includes hepatitis a virus cellular receptor 1 (encoding TIM-1), hepatitis a virus cellular receptor 2 (encoding TIM-3), and T cell immunoglobulin and mucin domain 4 (encoding TIM-4). Structurally, TIM proteins are type I membrane proteins characterized by an immunoglobulin variable (IgV) domain, a glycosylated mucin domain of variable length, and a single transmembrane domain. Notably, all TIM family members except TIM-4 possess a conserved tyrosine signaling motif in their cytoplasmic tail (58), which may contribute to their ligand recognition specificity (59).

TIM-3 interacts with four known ligands: carcinoembryonic antigen-associated cell adhesion molecule 1 (CEACAM1), high mobility group box 1 (HMGB1), phosphatidylserine (PtdSer), and galectin-9. The cytoplasmic tail of TIM-3 contains conserved tyrosine residues, including an Src homology 2 (SH2) binding motif (60). Galectin-9, the first identified TIM-3 ligand, is an S-type lectin abundantly expressed in hematopoietic cells. It binds to carbohydrate motifs on the IgV domain of TIM-3 through its two distinct carbohydrate-recognition domains, leading to Th1 cell apoptosis and inhibition of IFN-γ production (61).

Despite its role in T-cell suppression, TIM-3 exhibits atypical signaling mechanisms. Its cytoplasmic tyrosine residues interact with the proto-oncogene tyrosine-protein kinase Fyn and Human Leukocyte Antigen – B locus (HLA-B)-associated transcript 3 (BAT3) (62, 63). In the absence of ligand binding, BAT3 associates with TIM-3 cytoplasmic tail to maintain T cell activity (64). This unique regulatory mechanism suggests that TIM-3 modulates T-cell immunity through multiple molecular pathways (65).

In cancer research, TIM-3 has emerged as a critical immune checkpoint protein. Beyond its inhibitory effects on exhausted CD8+ T cells, TIM-3 plays a significant role in modulating Treg function within the tumor microenvironment. Notably, the majority of tumor-infiltrating Tregs express TIM-3 (55, 66), and antibody-mediated inhibition of TIM-3 in murine models has been shown to suppress tumor growth by reducing Treg effector molecule production (65).

The therapeutic potential of TIM-3 inhibition is currently being explored in clinical trials. To date, 33 monoclonal antibodies targeting TIM-3 have been developed and are being evaluated as monotherapies or in combination with other immune checkpoint inhibitors, chemotherapeutic agents, targeted therapies, or radiotherapy for their antitumor efficacy (67). These investigations underscore the growing interest in TIM-3 as a promising target for cancer immunotherapy.

### TIGIT

Currently, T-cell immunoreceptor with Ig and ITIM domains (TIGIT) is considered one of the most promising targets for cancer immunotherapy, with substantial evidence supporting its role in limiting both adaptive and innate immunity in tumors. TIGIT, also known as WUCAM, Vstm3, and VSIG9, is an immunoglobulin (Ig) superfamily receptor that plays a crucial role in restricting immune responses (68, 69).

Structurally, TIGIT comprises an intracellular short domain containing an immunoglobulin tyrosine tail (ITT)-like motif and an ITIM, an extracellular immunoglobulin variable domain, and a type I transmembrane domain (70, 71). TIGIT interacts with three ligands: CD155, CD112, and CD113, which are members of the NECL and connexin molecular families. Among these, CD155 is the primary ligand for TIGIT in both humans and mice (71). Crystal structure studies have shown that TIGIT and CD155 can form homodimers and heterotetramers during ligand-receptor interactions (72). Compared to CD155, TIGIT binds CD112 and CD113 less strongly (73, 74).

In addition to non-hematopoietic tissues such as the kidney, neurological system, and gut, CD155 is mostly expressed on dendritic cells (DC), T cells, B cells, and macrophages (75). In contrast, CD113 expression is restricted to non-hematopoietic tissues, including the placenta, testis, kidney, liver, and lung (76). CD112 is broadly expressed in both hematopoietic and non-hematopoietic tissues like bone marrow, kidney, pancreas, and lung (77). Notably, many malignant cancers overexpress CD155 and CD112. Tumor cells can be induced to overexpress these ligands by various stimuli, such as IFN-γ or oncogene expression (78).

TIGIT intracellular signaling domain is a key mediator of T and natural killer (NK) cell cytotoxicity. When CD155 binds to TIGIT, multiple signaling pathways are inhibited, and the SH2 domain-containing inositol 5-phosphatase (SHIP1) is recruited. This interaction phosphorylates the ITT-like motif and binds to Grb2, thereby suppressing downstream signaling (79). Premature binding of TIGIT to CD155 also inhibits the activation of extracellular signal-regulated kinase and mitogen-activated protein kinase kinase, which initiate the Mitogen-Activated Protein Kinase (MAPK) signaling cascade. Blocking the TIGIT signaling pathway restores Erk phosphorylation, and silencing SHIP1 reverses TIGIT/CD155-mediated inhibition, thereby restoring NK cell cytotoxicity (80). The sentinel antigen-presenting cells (APC) known as DC are in charge of antigen collection, movement, cytokine synthesis, and T and NK cell activation. However, mature dendritic cells are the only ones that can activate T cells, whereas immature dendritic cells may produce T cells that are unresponsive or resistant to immunotherapy. By activating CD155, TIGIT can cause DC to develop an immature tolerogenic phenotype, which leads to increased IL-10 secretion and decreased IL-12 production (71). Moreover, TIGIT is constitutively expressed on the majority of Treg and is essential for Treg maintenance and function.

In humans, follicular T helper cells, NK cells, regulatory T cells (Tregs), and activated CD8+ and CD4+ T cells all express TIGIT. TIGIT is not as well expressed in naïve T cells as DNAM-1/CD226 (81). TIGIT-blocking therapy holds promise, as intra-tumoral NK and T cells express higher levels of TIGIT than their peripheral blood counterparts (82). High TIGIT expression, a marker of exhausted NK and T cells, is associated with disease progression and immune evasion in various hematologic malignancies. Preclinical studies in hematologic cancers have further explored the therapeutic potential of anti-TIGIT agents (82). Additionally, TIGIT expression correlates with tumor stage, survival, and TIL composition in several malignancies (83).

There is growing evidence that TIGIT is highly expressed on TIL in different hematologic malignancies, leading to tumor progression and poor outcome. Furthermore, upregulation of TIGIT is associated with advanced disease stage (84, 85). Therefore, targeting TIGIT may be an effective approach for ICB therapy in hematologic malignancies. To date, immunotherapies targeting TIGIT have shown significant antitumor effects in several studies. In addition, dual blockade of TIGIT and PD-1 shows potential synergistic effects in enhancing antitumor immunity. For instance, studies in HL have revealed that 68-84% of T cells co-express TIGIT and PD-1 (86, 87). These findings suggest that combining anti-TIGIT and anti-PD-1/PD-L1 therapies may achieve more robust immune activation and tumor control. Currently, human anti-TIGIT monoclonal antibodies (mAbs) are being evaluated in Phase 1/2 clinical trials, both as monotherapy and in combination with anti-PD-1/PD-L1 antibodies or chemotherapy, for the treatment of malignant lymphomas. Collectively, these studies provide compelling support for the continued advancement of immunotherapies targeting the TIGIT axis in hematologic malignancies.

### A critical caveat for PD-1 blockade in T-cell malignancies

A notable adverse event during immune checkpoint inhibitor therapy is hyperprogressive disease (HPD) occurring shortly after the first administration of an ICI (88, 89). While HPD is a recognized entity in solid tumors, its occurrence in specific T-cell lymphomas is particularly concerning due to a distinct and paradoxical mechanism. Clinicians are well acquainted with HPD in patients with solid malignancies, though no unified definition exists (90–92). An acceptable definition involves a doubling or greater increase in tumor growth rate during immunotherapy. Existing evidence strongly suggests ICIs carry a significant risk of inducing HPD in specific T-cell lymphomas (particularly Adult T-cell Leukemia/Lymphoma (ATLL)-inactive subtype), potentially due to the PD-1 pathway’s unique “tumor-suppressive” function in these cancers (93). In this context, PD-1 signaling may act as an intrinsic brake on the proliferation and survival of the malignant T-cells themselves; thus, its blockade inadvertently releases this brake, leading to rapid tumor expansion. In principle, HPD patients should discontinue ICIs and pursue alternative salvage therapies. Some patients either enroll in clinical trials or receive best supportive care. The decision must be swift, as the clinical decline in these patients can be rapid. One study demonstrated that all ATLL patients treated with nivolumab exhibited rapid and significant disease progression, yet no unified pathophysiology could explain these events (94). Research indicates that in ATLL patients exhibiting this response to nivolumab, amplification of all major clones harboring diverse mutations suggests that rapid ATLL expansion following PD-1 blockade reflects an unexpected loss of ATLL suppression rather than a selective advantage for specific clones (95). This pattern of clonal evolution starkly contrasts with the selective outgrowth of a resistant subclone typically seen in other therapy-resistant cancers, reinforcing the model of a lifted inhibitory signal. Unexpectedly, clonal analysis revealed that ATLL clones were infected by Human T-cell Leukemia Virus type 1(HTLV-1) prior to completion of TCR gene rearrangement. Only a small fraction of HTLV-1-infected individuals develop ATLL, with the vast majority remaining asymptomatic throughout their lives (96). It remains unclear whether T-cell precursor infection and clonal expansion are associated with viral latency or pathogenesis, or whether they constitute a prerequisite for ATLL. Previous studies have demonstrated an increased incidence of collagen diseases among HTLV-1 carriers, including Sjögren’s syndrome, thyroid dysfunction, diabetes, and atherosclerosis (97). It is hypothesized that persistent HTLV-1 infection leads to altered immunoregulation. A systematic review and meta-analysis indicated that HTLV-1 infection itself has an adverse effect on overall survival (97). ATLL alone cannot fully account for the adverse impact of HTLV-1 infection on overall mortality, as its incidence is low; thus, HTLV-1-associated diseases collectively may contribute to poorer clinical outcomes (98). Although non-cancerous events associated with HTLV-1 infection are not fatal, they may diminish quality of life. Given that HTLV-1 carriers—the population at risk for ATLL—are present not only in endemic regions (e.g., southwestern Japan, the Caribbean, parts of South America) but also among immigrant populations in Europe and North America, clinicians must vigilantly assess HTLV-1 serostatus and consider regional epidemiology before initiating PD-1 inhibitor therapy. This pre-therapy screening is a critical safety measure that cannot be overemphasized.

It is crucial to note that HPD has been reported primarily with PD-1/PD-L1 inhibitors and not necessarily with other checkpoint agents like anti-CTLA-4, anti-LAG-3, or anti-TIGIT. This distinction may stem from the specific biology of PD-1 in T-cell lymphomagenesis, which is not shared by other checkpoint pathways. For instance, unlike PD-1, which can deliver a cell-intrinsic suppressive signal in malignant T-cells, CTLA-4 and LAG-3 primarily modulate early T-cell activation and dendritic cell function, respectively, rather than exerting a direct suppressive signal on the malignant T-cell clone itself. This mechanistic difference underscores why the HPD risk appears to be a unique vulnerability of PD-1/PD-L1 blockade in this specific oncological context.

Future advancements will involve novel monitoring algorithms integrating serial CT measurements or PET/CT with ctDNA to guide clinical decision-making and optimize ICI application. The use of ctDNA may be especially valuable for detecting the rapid clonal expansions characteristic of HPD even before radiographic progression is unequivocal, allowing for earlier intervention.

## PD-1 blockade in NK/T-cell lymphoma

Several tumors originating from mature T cells or NK cells are characteristically found in specific extranodal sites (99). Including extranodal NK/T-cell lymphoma (NKTCL), extranasal type (ENKTCL), primary intestinal T-cell lymphoma (ITCL), indolent gastrointestinal NK and T-cell lymphoproliferative disorders (LPDs), hepatosplenic T-cell lymphoma (HSTCL), and breast implant-associated anaplastic large cell lymphoma (BIA-ALCL). ENKTCL is the most common peripheral T-cell lymphoma in Asia.

PD-L1 expression is observed in 39% to 100% of NKTCL patients (100). Numerous ICIs have been studied for NKTCL, including the dual-targeting anti-PD1/PD-L1 antibody IBI318; the PD-1 mAbs pembrolizumab, sintilimab, tislelizumab, and toripalimab; and the PD-L1 mAbs sugemalimab and avelumab. The PD-1 inhibitor pembrolizumab was initially shown to be highly effective in treating relapsed or refractory NK/T-cell lymphoma by Kwong et al. Pembrolizumab, a PD-1 inhibitor, was added to the salvage treatment regimen for NK/T-cell lymphoma in the 2018 NCCN guidelines (101).

Pembrolizumab monotherapy, given at a dose of 2 mg/kg every three weeks, showed a 100% objective response rate (ORR) with no discernible toxicity in a retrospective analysis of seven patients with relapsed/refractory NKTCL. Interestingly, five patients (71%) experienced complete remission (CR), and all of them continued to have CR at the median 6-month follow-up (101). Pembrolizumab monotherapy, administered at a dose of 100 mg every three weeks, produced an ORR of 57% in another retrospective trial that included seven patients with recurrent NKTCL (102). Pembrolizumab monotherapy is being assessed in two ongoing trials (NCT04417166, NCT03728972) in patients with untreated early-stage NKTCL. Three patients who had previously failed L-asparaginase regimens were evaluated with nivolumab in one trial (103). All three patients in this trial showed initial responses; however, only one patient stayed in critical condition, the other two patients passed away from infections. In 28 patients with r/r NKTCL, the anti-PD-1 monoclonal antibody sintilimab monotherapy showed a 2-year overall survival (OS) rate of 78.6% and an ORR of 75.0% in the phase 2 single-arm ORIENT-4 study (104). Clinical trials for a number of additional ICIs are presently underway, including toripalimab, camrelizumab, tislelizumab, and IBI318. In a study that included nine patients with advanced NKTCL, PD-1 inhibitors in combination with P-GemOx chemotherapy showed an ORR of 88.9% and a CR rate of 77.8%, a 1-year PFS rate of 66.7%, and a 1-year OS rate of 100.0% (105). The combination of PD-1 inhibitors is being evaluated in a phase 2 trial (NCT04127227). A phase 2 trial (NCT04127227) is ongoing to evaluate PD-1 inhibitors in combination with P-GemOx chemotherapy for advanced NKTCL patients. The anti-PD-L1 monoclonal antibody avelumab, when used as monotherapy, showed an ORR of 38% and a CR of 24% in a phase 2 trial that included 21 patients with r/r NKTCL (106). Respondents in this experiment, however, showed a comparatively lengthy duration of response, with the longest lasting more than 25 months (106). Additionally, the study found a positive relationship between treatment response and PD-L1 expression, indicating that measuring PD-L1 expression levels could help identify individuals who are more likely to benefit from PD-L1 inhibitors. The anti-PD-L1 monoclonal antibody sugemalimab showed an ORR of 46.2% and a CR rate of 30.4% in a single-arm phase II trial with 80 patients with r/r NKTCL. The OS rate was 68.6% at one year and 54.6% at two years (107).

Collectively, the data solidifies the role of ICIs in r/r NKTCL. However, a deeper analysis uncovers critical nuances. First, heterogeneity in efficacy exists among different PD-1/PD-L1 inhibitors, potentially due to distinct drug properties or patient populations. The positive correlation between PD-L1 expression and response is pivotal, suggesting PD-L1 as a predictive biomarker for future precision medicine strategies. Second, combination therapy represents a clear path to enhanced efficacy. The superior outcomes from combining a PD-1 inhibitor with chemotherapy underscore their synergistic potential, likely through chemotherapy-induced immunogenic cell death.

The safety profile of PD-1 inhibitors is also a matter of concern. Common adverse reactions to PD-1 inhibitors include fatigue, fever, chills, and infusion reactions. Organ-specific adverse reactions encompass pruritus, rash, colitis, immune-mediated pneumonia, hepatitis, and others. Although adverse reactions to PD-1 inhibitors occur less frequently, they warrant close attention from clinicians.

## ICIs combined with other treatment regimens

ICIs, one of the most well-known developments in the field of tumor immunotherapy, have produced notable therapeutic results due to their long-lasting antitumor effects and expansive range of biological activity in a variety of histological tumor types (108, 109). However, only a small percentage of patients respond to these drugs (110). There are too many resistance mechanisms, including immunosuppressive TMEs, gut microbiome abnormalities, epigenetic changes, and activation of other immune checkpoints. Combination therapy tactics are gaining popularity in the battle against drug resistance because they activate several anti-tumor immune mechanisms (111). Numerous studies have demonstrated that ICI can effectively prevent tumor resistance to ICI therapy when used in conjunction with other treatments such chemotherapy, radiation, cancer vaccines, and anti-angiogenic drugs (112–115) (**Table 2**).

**Table 2:** Key combination studies with immune checkpoint inhibitors in lymphoma.

Disease	ICP	Type of combination therapy	Clinical benefits	Adverse event	NTC number	Phase
Relapsed or refractory HL	PD-1(nivolumab/pembrolizumab)	Radiotherapy	ORR 100% CR 58%	The majority of AEs were grade 1-2, mainly related to radiotherapy, and all resolved.	NCT04419441	Retrospective
Recurrent Hodgkin lymphoma after PD1 treatment	PD-1(nivolumab)	Radiotherapy	ORR 37.5% CR 4% PR 33% 12 months PFS 29.2% 18 months PFS 12.5% 12 months OS 100% 18-month OS 76%	Rkin, mucosal, reactions	NCT03480334	Phase 2
Diffuse large B cell lymphoma or grade 3b follicular lymphoma	PD-1(pembrolizumab)	Radiotherapy	5-year PFS 71% 5-year OS 83%	Rash, thyroiditis, rheumatoid arthritis	NCT02541565	Phase 2
Relapsed or refractory advanced follicular lymphoma	PD-L1(atezolizumab)	Low-dose localized radiotherapy	NA	NA	NCT03465891	Phase 2
Advanced lymphoma	PD-1(pembrolizumab)	Radiotherapy	NA	NA	NCT02408042	Phase 2
Relapsed/refractory gray area lymphoma	PD-L1(tirelizumab)	Chemotherapy(ICE)	NA	NA	NCT04860674	Phase 2
Relapsed or Progressive Classic Hodgkin Lymphoma	PD-1(pembrolizumab)	Chemotherapy(Azacitidine)	NA	NA	NCT05355051	Phase 2
Relapsed/refractory Hodgkin lymphoma	PD-1(pembrolizumab)	Chemotherapy(DHAP)	NA	NA	NCT04091490	Phase 2
First relapsed or refractory classical Hodgkin lymphoma	PD-1(pembrolizumab)	Chemotherapy(ICE or DHAP)	NA	NA	NCT04838652 (Phase 2)	Phase 2
Patients with relapsed/refractory Hodgkin lymphoma	PD-1(nivolumab)	Chemotherapy(Bendamustine)	ORR 87% CR 57% PR 30% 2-year OS 96.7% 2-year PFS 23.3%	Infections	NCT0334365	Phase 2
Untreated B-cell lymphoma	PD-1(pembrolizumab)	Chemotherapy(R-CHOP)	5-year PFS 71% 5-year OS 83%	Rash, thyroiditis, rheumatoid arthritis	NCT03995147	Phase 2
Classic Hodgkin lymphoma	PD-1(low-dose nivolumab)	Chemotherapy(AVD)	CR 65% PR 10% PD 5%	Neutropenia, hypothyroidism	NCT05772624	Phase 2
Classic Hodgkin Lymphoma	PD-1(nivolumab)	Chemotherapy(Vinbla stin)	NA	NA	NCT03580408	Phase 2
Relapsed or refractory Hodgkin lymphoma	PD-1(pembrolizumab)	Chemotherapy(ICE)	CR 86.5% ORR 97.3% PR 10.8% 2-year PFS 87.2% 2-year OS 95.1%	NA	NCT03077828	Phase 2
Relapsed or refractory Hodgkin lymphoma	PD-1(pembrolizumab)	Chemotherapy(GVD)	ORR 100% CR 95%	Transaminitis, neutropenia, mucositis, thyroiditis, rash	NCT03618550	Phase 2
High-risk diffuse large B-cell lymphoma	PD-L1(durvalumab)	Chemotherapy(R-CHOP (± lenalidomide)	CR 67%	Fatigue, neutropenia, neuropathy, nausea, diarrhea, fever	NCT03003520	Phase 2
Relapsed or refractory Hodgkin lymphoma	PD-1(nivolumab)	Targeted CD30(Brentuximab Vedotin)	ORR 85% CR 67% 3-year OS 93%	Nausea, infusion-related reactions, systemic corticosteroid therapy.	NCT02572167	Phase 2
Relapsed or Refractory Classical Hodgkin Lymphoma Previously Treated with Brentuximab Vedotin or Checkpoint Inhibitors	PD-1(nivolumab)	Targeted CD30(Brentuximab Vedotin)	NA	NA	NCT05039073	Phase 2
Relapsed or treatment-naïve peripheral T-cell lymphoma	PD-1(pembrolizumab)	Targeted HDAK(Romidepsin)	ORR 50% CR 35% PR 14%	Nausea, vomiting, fatigue cytokine storm, gastritis, colitis, and pneumonia	NCT03278782	Phase 2
Primary mediastinal large B-cell lymphoma	PD-1(nivolumab)	Targeted CD30(Brentuximab Vedotin)	ORR 73.3% 24 months PFS 55.5% 24 months OS 75.5%	Neutropenia	NCT02581631	Phase 2
Recurrent peripheral T-cell lymphoma	PD-1(nivolumab)	Targeted CD30(Brentuximab Vedotin)	NA	NA	NCT04795869	Phase 2
Recurrent or refractory diffuse large B-cell lymphoma or primary mediastinal large B-cell lymphoma	PD-1(nivolumab)	Targeted PI3k(Copanlisib Hydrochloride)	NA	NA	NCT03484819	Phase 2
Hematologic malignancies	PD-L1(atezolizumab)	Targeted ACP-196 (Acalabrutinib)	ORR 38.5%	Diarrhea, fatigue, headache, cough, nausea,	NCT02362035	Phase 1/Phase 2
Relapsed or refractory non-Hodgkin lymphoma	Pd-L1(atezolizumab)	Targeted CD20(Obinutuzumab)	NA	NA	NCT03369964	Phase 1
Relapsed or refractory non-Hodgkin lymphoma	PD-1(pembrolizumab)	Targeted BTK(Ibrutinib)	NA	NA	NCT02950220	Phase 1
Follicular lymphoma	PD-1(nivolumab)	Targeted CD20(Rituximab)	ORR92% CR 59% 4-years PFS 58%	Elevated amylase/lipase, liver enzyme derangement, infection	NCT03245021	Phase 2
Relapsed or refractory classical Hodgkin lymphoma	PD-1(pembrolizumab)	Targeted CD47(Magrolimab)	NA	NA	NCT04788043	Phase 2
Refractory or recurrent B-cell lymphoma	PD-1(atezolizumab)	Targeted CD40 Ab(Selicrelumab)	NA	NA	NCT03892525	Phase 1
Relapsed or refractory T-cell lymphoma	PD-1(pembrolizumab)	Targeted CD30(brentuximab vedotin)	NA	NA	NCT05313243	Phase 2
Relapsed or Refractory Diffuse Large B-Cell Lymphoma	PD-1(pembrolizumab)	Targeted HDAK(CXD101)	NA	NA	NCT03873025	Phase 1/Phase 2
Relapsed or refractory classical Hodgkin lymphoma	PD-1(nivolumab)	ICI(Ipilimumab)	NA	NA	NCT04938232	Phase 2
Classic Hodgkin lymphoma.	PD-1(pembrolizumab)	ICI(Favezelimab)	ORR 83% CR 37% PR 47% 24-month PFS 46% 24-month OS 93%	Hypothyroidism infusion related reactions, fatigue, colitis, pneumonia, severe skin reactions, hepatitis	NCT03598608	Phase 1/Phase 2
Relapsed/refractory diffuse large B-cell lymphoma	PD-1(pembrolizumab)	CD-19 CAR-T(Tisagenlecleucel)	CR33% PR16% PD50% ORR 50%	Febrile, neutropenia, CRS	NCT03630159	Phase 1b
Stable/progressive DLBCL	PD-1(nivolumab)	CD-19 CAR-T	ORR 84% CR 53% 6-month PFS 50% 12-month PFS 42% 6-month OS 85% 12 months OS 51%	Cytopenia, CRS	NCT05385263	Phase 2
Relapsed/refractory B-cell non-Hodgkin lymphoma	PD-1(tislelizumab)	CD19/22 CART	ORR 87% CR 68% OS 81.3% 1-year PFS 68%	CRS, fatigue, fever, hematologic toxicity, neutropenia thrombocytopenia, anemia, low neutrophil, platelet count	NCT04539444	Phase 2
Primary mediastinal B-cell lymphoma	PD-1(pembrolizumab)	CAT-T	NA	NA	NCT05934448	Phase 2
Follicular lymphoma	PD-1(pembrolizumab)	Neo Vax	NA	NA	NCT03361852	Phase 1

The application of ICIs represents a landmark advancement in lymphoma treatment, achieving breakthroughs as monotherapies in subtypes with specific immune microenvironment characteristics, such as classical Hodgkin lymphoma. However, objective response rates for ICI monotherapy remain unsatisfactory in most lymphoma subtypes. This challenge stems from the fact that a complete antitumor immune response is a multi-step process, and tumor cells can exploit multiple mechanisms to disrupt each stage. Single PD-1/PD-L1 blockade can only relieve suppression at the effector T cell stage but fails to effectively address upstream issues such as deficient tumor antigen presentation, insufficient T cell infiltration, or immunosuppressive microenvironments. This review outlines the therapeutic advantages of ICIs as a pioneering strategy in tumor immunotherapy and discusses recent reports evaluating their combination with other immunotherapies. The synergistic use of ICIs alongside chemotherapy, radiotherapy, targeted therapies, and other modalities aims to jointly initiate, amplify, and sustain the entire immune cycle. By combining ICI with other modalities such as chemotherapy, radiotherapy, and targeted therapy, the goal is to identify the optimal treatment regimen.

### Combination with chemotherapy

**Figure 2:**
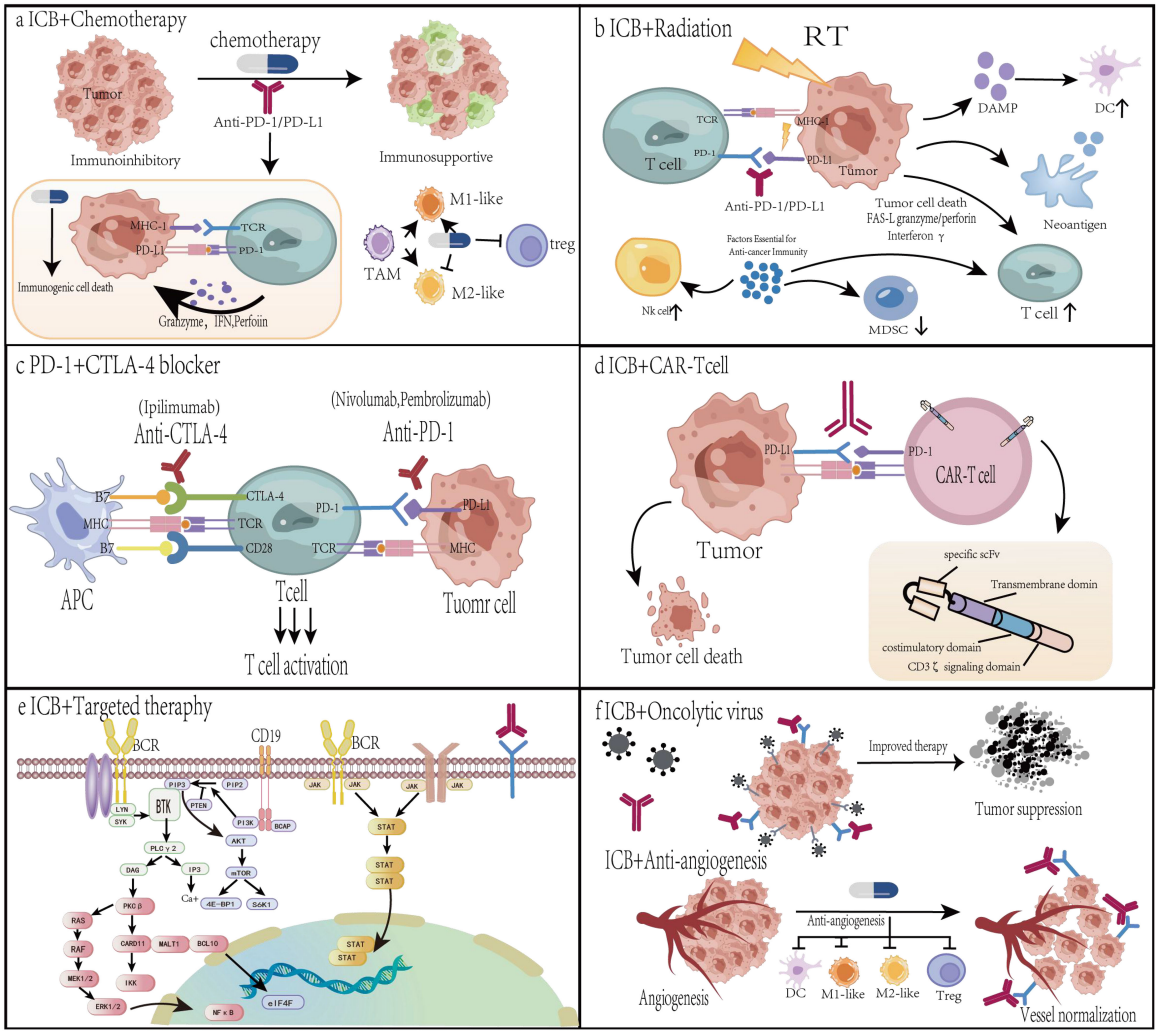
The mechanisms behind the synergistic anticancer effects of PD-1/PD-L1 in conjunction with chemotherapy, radiation, or angiogenesis inhibitors. **(a)** Combined with chemotherapy. Some cytotoxic chemotherapy drugs can cause immunogenic cell death and boost anti-tumor immunity. Immunogenic cell death is distinguished by a number of up-regulated damage-associated molecular patterns (DAMPs). I addition to immunogenic cell death, low-dose chemotherapy depletes regulatory T cells (Tregs) and promotes repolarization of tumor-associated macrophages (TAMs) from M2 to M1 phenotypes. **(b)** Radiotherapy enhances the effectiveness of α-PD-1/PD-L1 treatment. First, radiation causes immunogenic cell death, boosts anti-tumor immune responses, encourages T-cell infiltration, and increases the T-cell receptor (TCR) pool in the tumor microenvironment. Radiotherapy increases the expression of PD-L1 on tumor cells, which can be used to target more α-PD-1/PD-L1. **(c)** Combination therapy with PD-1 and CTLA-4 blockers. Antibodies against PD-1/PD-L1 and CTLA-4 can inhibit their activation, respectively. A combination of PD-1 and CTLA-4 inhibitors may have a synergistic impact. **(d)** Combination with CAR-T. Collection of blood and isolation of leukocytes via leukapheresis, genetic alteration of T cells, multiplication of the resulting modified CAR-T cells, injection of CAR-T cells, and mAb inhibition of inhibitory signaling pathways to improve therapeutic efficacy. **(e)** In combination with targeted therapy. Oncogenic pathways, such as MAPK and PI3K-AKT, stimulate PD-L1 transcription. Targeted therapies such as EGFR-TKI, ALK-TKI, and RAS inhibitors not only directly limit tumor growth but also lower intrinsic PD-L1 levels. **(f)** In conjunction with oncolytic viruses or angiogenesis inhibitors. When infected with oncolytic viruses, tumor cells launch an antiviral response by generating antiviral cytokines. Lysis of tumor cells results in the release of viral progeny, damage-associated molecular patterns, pathogen-associated molecular patterns, and tumor-associated antigens. Viral offspring infect more tumor cells. Angiogenesis inhibitors suppress pro-angiogenic pathways, promote vascular normalization, increase tumor perfusion and oxygenation, treat hypoxic TME, and improve medication delivery.

Even though chemotherapy is typically not curative, it is nevertheless the cornerstone of cancer treatment. Historically, most chemotherapeutic agents were developed based solely on their direct cytotoxic effects, without considering their impact on the immune system (116). Tumor shrinking is one of the primary advantages of cytotoxic chemotherapy. One of the primary benefits of cytotoxic chemotherapy is tumor shrinkage. Tumor cells are a major source of immunosuppressive tumor microenvironments (TMEs). Furthermore, losing tumor cell quantity reduces the amount of cancer cells that immune cells must remove. This can be a startling result, especially in tumors with minimal TME immune cell invasion (117). Some cytotoxic chemotherapy drugs can cause immunogenic cell death and boost anti-tumor immunity (**Figure 2a**). In immunocompetent hosts, immunogenic cell death (ICD) is a highly orchestrated form of cell death that triggers adaptive immune responses (118). Numerous studies have demonstrated that cytotoxic chemotherapy improves immunotherapy and causes ICD (115). Interactions between cytotoxic chemotherapy and immunotherapy augmentation have been demonstrated in numerous investigations. Following the advancement of their cancer on anti-PD1 therapy, some individuals with chemotherapy-resistant malignancies respond to a second round of chemotherapy.

However, one of the main negative effects of chemotherapy on the immune system is the depletion of lymphocytes, which may have immunosuppressive effects. Some immunosuppressive medications used for autoimmune diseases are actually cytotoxic chemotherapeutic agents that are employed in cancer treatment, albeit at different doses. The impact of chemotherapy-induced lymphocyte depletion on immune suppression remains a topic of debate. Additionally, chemotherapy may impair tertiary lymphoid structures (TLS), which are ectopic lymphoid tissues that develop in non-lymphoid organs, including tumors, and resemble lymph nodes (119, 120). Research indicates that TLS can attract lymphocytes to tumors and enhance immune responses against cancer. Moreover, the lymphocyte-depleting effects of chemotherapy may influence TLS function due to the direct cytotoxic effects of these drugs (117).

Additionally, chemotherapy induces CXCL10 to be generated locally, which draws T cells to the tumor bed and stimulates the formation of tumor-specific CTLs (121). Chemotherapy reduces the amount of immunosuppressive cells, such as myeloid-derived suppressor cells (MDSCs) and Treg (122). Chemotherapy-induced tumor shrinking minimizes the risk of drug-resistant clones while also prolonging the efficacy of immunotherapy. Several chemotherapeutic drugs have shown the capacity to alter several anti-cancer immune pathways within the last ten years (123). Given the widespread use of chemotherapy to alter the tumor immune response, combination therapy including ICIs and chemotherapeutic medications significantly enhances clinical outcomes by raising CTL activity. Chemoimmunotherapy has been used in several clinical trials for almost all major malignancies, and these trials have been approved by the FDA.

The combination of immune checkpoint inhibitors and chemotherapy has emerged as a promising therapeutic strategy in the treatment of lymphomas. ICIs, such as anti-PD-1 and anti-CTLA-4 antibodies, have demonstrated significant efficacy in enhancing anti-tumor immune responses by blocking inhibitory signals that suppress T cell activation. However, the response rates to ICIs alone are often limited in certain types of lymphomas. The integration of these two modalities leverages the tumor-shrinking effects of chemotherapy to enhance the efficacy of ICIs, while ICIs can augment the immune system ability to recognize and eliminate residual cancer cells. This synergistic approach has shown encouraging results in clinical trials, with improved overall response rates and progression-free survival in patients with relapsed or refractory lymphomas (**Table 2**).

Combining ICI with traditional chemotherapy—particularly in patients with HL who have undergone multiple treatments—yields superior efficacy and safety (124), in contrast to its limited application in non-Hodgkin lymphoma. The NCT0334365 study evaluated the safety and efficacy of natalizumab and bendamustine (NB) in patients with relapsed or refractory Hodgkin lymphoma who had previously failed nivolumab monotherapy. The study demonstrated that the NB combination achieved high short-term response rates and overall survival in this patient population. However, long-term disease control was limited, indicating that most patients ultimately required additional therapy to sustain efficacy (125). Rituximab, cyclophosphamide, doxorubicin, vincristine, and prednisone (R-CHOP) have long been the standard first-line treatment for DLBCL. Despite its widespread use, up to 40% of patients experience relapse, and efforts to improve upon the R-CHOP regimen have largely been unsuccessful. The incorporation of immune checkpoint inhibitors (ICIs) into the R-CHOP regimen has shown promise, achieving durable remissions in the majority of patients, particularly those with PD-L1–expressing disease (126). Preliminary results from the NCT05772624 trial showed significant efficacy of low-dose nabulizumab in combination with AVD in the first-line treatment of classical Hodgkin lymphoma, with all patients achieving an objective response within two cycles. This regimen is not only effective, but also has low economic and biological toxicity, making it suitable for replication in resource-limited areas (127). Combination therapies show promise in both first-line and salvage treatments for lymphoma, though their objectives and challenges differ. First-line therapy aims to improve cure rates, while salvage therapy seeks to overcome drug resistance and extend survival opportunities for patients. Despite impressive short-term response rates, long-term disease control remains a common challenge for combination therapies, as demonstrated by studies like NCT0334365. Current data suggest that first-line therapies (e.g., NCT05772624) appear to yield a higher likelihood of durable remission compared to salvage therapies; however, this requires longer follow-up to confirm.

ICIs combination chemotherapy as a strategy still faces challenges. Ongoing clinical trials are further exploring the synergistic potential of this combination therapy with the aim of establishing a more effective and personalized treatment approach for lymphoma patients.

### Combination with radiotherapy

Radiation therapy (RT) is administered to over half of cancer patients. Through a number of processes, including necrosis, autophagy, and apoptosis, RT can directly cause cancer cell death (128). Furthermore, inflammatory mediators that are generated by dying cells that have been exposed to radiation might draw in and alter immune cells in the tumor microenvironment in order to further destroy cancer cells. Radiation causes immunogenic cell death, boosts anti-tumor immune responses (**Figure 2b**). In addition to changing the local TME, RT improves adjuvant and antigenic qualities. RT increases tumor antigenicity in a number of ways. First, radiation improves the presentation of tumor antigens and causes MHC-I expression (129). Second, ICD is brought on by radiation. Membrane-bound protein A1 guides antigen-presenting cells toward cancer cells that are dying during ICD, while chemicals like High-Mobility Group Box 1 (HMGB1), HSP70, and HSP90 encourage T cells to absorb and deliver tumor antigens. Research has demonstrated that radiation triggers the release of HMGB1 (130) and the translocation of calmodulin to the plasma membrane (131). Third, radiation increases the uptake and antigen presentation of cancer cells while suppressing the expression of CD47 on the cell surface (132). Many cancer cells overexpress CD47, which tells the APC, “don’t eat me” (133). Fourth, ionizing radiation produces reactive oxygen species (ROS), which can alter macromolecules like DNA and proteins and make them more antigenic. Radiation-induced tissue damage is dependent on both direct DNA damage and the generation of ROS and oxygen (134). Increased adjuvanticity is another significant way that radiation contributes to anticancer immunity. By upregulating the expression of the type I interferon pathway, the cyclic GMP-AMP synthase (cGAS)/Stimulator of Interferon Genes (STING) system triggers both innate and adaptive immune responses in response to radiation-induced DNA damage and cytoplasmic leaking of micronucleated DNA. Radiation-induced antitumor immunity depends on this mechanism. T cell initiation is hampered when cGAS is silenced in dendritic cells originating from bone marrow (135). Mitochondrial DNA breaks work in concert with nuclear DNA breaks to trigger the type I interferon response (136). The release of ICDs, damage-associated molecular patterns (DAMPs), and cytokines can decrease immunosuppressive cells, increase adjuvanticity, induce migration of pro-cancer immunological subpopulations, modify the TME, and shift the immune response to cancer cell death in addition to the cGAS-STING pathway. In general, radiation triggers anticancer immunity by transforming cancer cells into *in situ* vaccines.

Radiation therapy can both cure cancer patients and harm healthy tissues. When the radiation dose is greater than what the surrounding normal tissues can heal, irreversible damage results. Numerous investigations have demonstrated that radiation stimulates MDSC infiltration and aggregation and supports immunosuppressive TME via a variety of mechanisms (137). Furthermore, radiation increases the production of transforming growth factor-β (TGF-β) and TGF-β family activator A, which decreases -CD8 + T cell infiltration and increases -Treg cell recruitment (138). Following radiation, there was an increase in TGF-β expression (139). In summary, radiation-induced immunosuppressive TME production is a complicated process, and focusing on these immunosuppressive elements offers a fresh approach to boosting RT-induced antitumor immunity. Therefore, improving the accuracy and reducing the dose of radiotherapy without compromising the efficacy of radiotherapy is the main goal of current research.

In both animal models and patients with solid tumors, positive interactions between radiation and immunotherapy have been shown. The so-called “tumor vaccine in situ” is the result of radiotherapy induction of immunogenic cell death, which is linked to the release of antigens, the creation of cytokines, and the activation of complement (140, 141). Following local radiotherapy, all of these interactions strengthen and promote the systemic immune response (142). These pathways demonstrate the various ways in which radiation therapy and the immune system interact, potentially resulting in a systemic immunological response following local irradiation (143).

As a novel treatment approach, combined radiation and immunotherapy has shown promising clinical outcomes. The effectiveness of combination therapy with immunotherapy and radiotherapy (also known as radiation splicing) has been examined in more than 100 clinical trials to date (**Table 2**). The cell adhesion factors intercellular adhesion molecule-1 (ICAM-1) and vascular cell adhesion molecule-1 (VCAM-1) on the surface of cancer cells have been demonstrated to be markedly up-regulated by radiation therapy. In TME, anti-PD-1 antibodies and radiation therapy work together to stimulate tumor-specific T cells (144). Furthermore, anti-PDL 1 antibody and radiation therapy enhanced CD 8 + T cell infiltration and reduced MDSC and regulatory T cell accumulation, thereby enhancing anti-tumor immunity (145). Increased immunotherapy produces better rates of local control at the same radiation dose, according to preclinical research (146). In NSCLC, radiotherapy stimulates immunotherapy by activating specific club cell types, according to a recent preclinical study. By successfully suppressing MDSC, these cells subsequently release proteins that greatly increase the effectiveness of PD-1 inhibitors in reducing inflammation and boosting the immune response against tumors (147).

Combining ICIs may enhance the efficacy of ICIs against lymphoma while preserving the development of immune responses (124). In the context of cHL, which is known to be highly sensitive to radiotherapy, this combination may exhibit significant synergistic effects and enable therapeutic strategies using lower radiation doses than those employed in current regimens. An open-label, single-arm phase II study assessed the safety and efficacy of sequential hypofractionated radiotherapy followed by zimberelimab and R-GemOx (rituximab, gemcitabine, oxaliplatin) in patients with primary refractory DLBCL (148). RT doses of 36 and 24 Gy were delivered to the gross and target volumes in 12 fractions, followed by zimberelimab and R-GemOx. The overall response rate within the irradiated field was 92.3%, and a complete response (CR) was achieved by 61.5% of patients; however, 38.5% experienced disease progression. This study demonstrates the favorable efficacy of radiotherapy combined with immunochemotherapy in refractory DLBCL. Compared to the 30-40% CR rate of conventional chemotherapy regimens, this regimen achieves a complete remission rate exceeding 50%. Although radiotherapy combined with immune checkpoint inhibitors has demonstrated efficacy unseen in traditional regimens, the current challenge lies in determining whether radiotherapy should precede immunotherapy or be administered concurrently. The optimal sequencing remains unknown. Concurrent administration may yield the strongest synergistic effect but could also increase toxicity at specific sites. Additionally, should high-dose, fractionated SBRT be employed to maximize immunogenic cell death, or should a more conventional fractionation schedule be used? The optimal radiotherapy regimen has yet to be determined. Further research is still needed in this field.

### Combined with another immune checkpoint inhibition

CTLA-4 and PD-1 blockers have shown remarkable sustained response rates, longer survival times in responding patients, and a tolerable toxicity profile as new inhibitory receptors are investigated as potential immunotherapy targets (149–151). However, low response rates—just a tiny portion of patients react to treatment—limit the advantages of monotherapy (150). Thus, to increase patient response rates and survival, CTLA-4 and PD-1 blocking combos have been suggested (**Figure 2c**). Blocking CTLA-4, which is mainly involved in controlling T cell activation in lymph nodes and tissues and preventing DC activity through Treg cells, is believed to work in tandem with blocking PD-1, which is mainly involved in preventing effector T cell and NK cell activation in peripheral tissues and inducing Treg cell differentiation (152, 153). Combination therapy including CTLA-4 and PD-1 inhibitors is frequently employed in clinical settings (154). With the help of CTLA-4 inhibitors, T cell activity can be restored by blocking the potent inhibitory signals that CTLA-4 produces to stop T cell proliferation and activation. Therefore, the primary function of CTLA-4 is to signal interactions between lymphocytes. PD-1 prevents the immune response from being activated (155).

For certain kinds of lymphoma, combination therapy with PD-1 and CTLA-4 receptor blockers has also been beneficial. Ipilimumab and nivolumab were used in study NCT02304458 to treat sarcomas or solid tumors that had relapsed or were refractory (156). Out of the 10 Hodgkin lymphoma patients who could be evaluated for response, one experienced a full remission, two experienced a partial remission, five experienced stable disease (median 12–8 cycles, IQR 6-18), and two experienced a mixed response, meaning that while the size of the target lesion decreased, a new lesion appeared during the study period. The response rate seems to be lower than the overall response rate of 66–87% observed in adult Hodgkin lymphoma patients, despite this encouraging activity (156). In a multicohort phase 1/2 study assessing the anti-LAG-3 monoclonal antibody favezelimab in combination with pembrolizumab in the treatment of R/R hematologic malignancies (NCT03598608) (157), a cohort of patients with anti-PD-1 primary R/R cHL (cohort 1) showed sustained antitumor activity and a tolerable safety profile. Currently, multiple novel drugs or combination strategies targeting TIGIT and PD-1/PD-L1 co-inhibition are undergoing evaluation in clinical trials (158). Co-inhibition of TIGIT and PD-1/PD-L1 can synergistically induce tumor rejection and has been approved in clinical trials, offering new options for cancer immunotherapy. Although the optimal combination strategies and patient selection criteria remain under investigation, this approach represents a promising pathway for developing more effective cancer immunotherapies. Combining additional ICIs is seen as a viable treatment approach since inhibition of TIGIT and CTLA-4 offers substantial therapeutic benefits. Dual blockage of immunological checkpoints has been shown in some studies to significantly increase patient response and survival rates, which is a breakthrough in immunotherapy (111). Adverse events linked to the immune system, however, are crucial to combination therapy effectiveness. Research should shift its focus to managing and forecasting these occurrences.

### Combination with CAR-T therapy

Chimeric antigen receptor (CAR) T-cells are synthetically engineered T-cells that express CARs with target specificity to bind antigens in an MHCC-independent manner (159). In order to destroy malignancies, CAR-T cell treatment involves genetically modifying a patient T cells to express specific CARs, which are subsequently ex vivo grown and reinfused into the patient (**Figure 2d**). CARs are synthetic receptors made up of an intracellular T-cell activation and costimulatory signaling domain (CD3ζ, CD28, and/or 4-1BB), a transmembrane structural domain, and an extracellular structural domain (scFv) derived from an antibody reactive to a tumor antigen (160). T cells for antigen-unrestricted cytokine-initiated kill (TRUCK), a fourth-generation CAR, encode suicide genes to avoid toxicity or cytokine-producing genes to increase CAR-T activity (160). Axicabtagene ciloleucel (axi-cel, CD3ζ-CD28) and Tisagenlecleucel (tisa-cel, CD3ζ-41bb) are two second-generation CAR-T cell treatments that were authorized by the US FDA in 2017 (161, 162).

The primary cause of relapse or lack of response following CAR T-cell therapy is still poor T-cell persistence, even with the promising outcomes of anti-CD19 CAR T-cell therapy. In actuality, the CR rate for anti-CD19 CAR T cell therapy is just 29% for Chronic Lymphocytic Leukemia (CLL) patients (163) while the CR rate for Acute Lymphoblastic Leukemia (ALL) patients is 90% (164). Notably, responding patients had a decreased percentage of PD-1-expressing CAR T cells, but non-responding CLL patients showed transcriptional activation of genes linked to apoptosis and depletion (165). Furthermore, negative responses were associated with co-expression of PD-1 on CAR T cells with either LAG-3 or TIM-3 (165), underscoring the crucial function of T cell immune checkpoint molecules in regulating CAR T cell activity. Although an adequate safety profile has been documented for this combination therapy thus far, the therapeutic advantage of combining PD-1/PD-L1 inhibitors with CD19 CAR T cell therapy has not been shown (166). The safety of CD19 CAR T-cell treatment in conjunction with PD-1 blocking was further demonstrated in a recent trial involving 14 children with B-ALL who had undergone numerous previous therapies. PD-1 blockade may prolong CAR-T cell activity, as evidenced by the fact that 3 out of 6 patients treated with PD-1 inhibitor CAR T cell therapy reconstituted B cell regeneration diseases (167). Combining PD-1 blockage with inhibitory molecules like TIM-3 and LAG-3 may enhance CAR T cell performance even more, though proof of concept requires more research.

The management of patients with relapsed or refractory hematologic malignancies, particularly those with R/R B-NHL after multiple lines of therapy, remains a significant challenge for clinicians. In this context, the combination of CAR-T therapy with ICIs is considered a promising future therapeutic opportunity. In September 2021, a Phase I/IIA clinical trial (168) demonstrated improved clinical efficacy when pembrolizumab (a PD-1 inhibitor) was administered following CAR-T cell infusion in patients with refractory B-cell lymphoma. Among the 12 evaluated patients, one achieved complete remission and two achieved partial remission. The study also demonstrated increased activation and proliferation of CAR-T cells. This suggests the potential benefit of combination therapy in improving objective response rates in advanced patients (169, 170). However, this combination strategy has several limitations. First, the blocking effect of PD-1 inhibitors is transient, requiring repeated administration. Second, PD-1 inhibitors can be captured by TAMs before reaching the CAR-T cell surface, thereby eliminating their ability to block the PD-1/PD-L1 pathway. Third, systemic administration of ICIs induces significant systemic side effects. In short, these limitations pose significant obstacles that must be overcome to unlock the potential of this combined strategy fully.

T-cell/Histiocyte-Rich Large B-Cell Lymphoma (THRLBCL) represents a rare and aggressive subtype of lymphoma, characterized by a unique tumor microenvironment where malignant B-cells are sparse and embedded within a dense background of non-neoplastic T-cells and histiocytes. Although CAR-T cell therapy has demonstrated success across various lymphoma subtypes, THRLBCL presents a unique challenge due to its high rate of treatment resistance (171). The efficacy of standalone CAR-T cell therapy for THRLBCL is significantly less impressive (171). The largest real-world study to date, encompassing R/R THRLBCL patients treated with commercial CAR-T products, reported a 100-day ORR of 50% and a complete response CR rate of only 28% (172). This suboptimal performance, particularly the low CR rate which is a critical predictor of long-term survival, underscores a significant unmet clinical need and highlights THRLBCL as a “difficult-to-treat” subtype even with advanced cellular therapies. Overcoming resistance mechanisms remains critical for enhancing the therapeutic efficacy of CAR-T cell therapy in THRLBCL. Combining CAR-T cell therapy with immune checkpoint inhibitors (ICIs) emerges as a promising strategy. By targeting the PD-1/PD-L1 pathway, ICIs can potentially counteract the immunosuppressive microenvironment and enhance CAR-T cell function (171). Pioneering clinical trials are now actively exploring this combination. For instance, the study NCT05934448 is specifically investigating the safety and efficacy of anti-PD-1 therapy administered following CD19 CAR-T treatment in patients with R/R B-cell lymphomas, including THRLBCL. The primary objectives will focus on managing the unique safety profile of this combination, particularly the risks of overlapping toxicities such as heightened cytokine release syndrome (CRS) or immune effector cell-associated neurotoxicity syndrome (ICANS). Key exploratory endpoints will include longitudinal monitoring of CAR-T cell persistence and exhaustion markers, as well as deep immune profiling of the TME pre- and post-therapy to validate the mechanistic hypothesis. Future breakthroughs in this combination therapy will first hinge on optimizing the timing of PD-1 blockade and CAR-T infusion. Secondly, biomarkers predictive of treatment efficacy must be identified to enable precise patient selection, ultimately guiding personalized treatment strategies. The evolving exploration of CAR-T therapy in combination with ICIs is shedding new light on strategies to overcome treatment resistance in THRLBCL. This approach signifies a paradigmatic shift in the management of refractory hematologic malignancies. Notably, early-phase clinical trials—such as NCT05934448—are pioneering in their efforts to dissect the intricate interplay between cellular therapeutics and the tumor microenvironment. If this combinatorial approach is successful, it could have an impact that goes far beyond THRLBCL and provide a potentially revolutionary therapeutic framework for a wider range of malignancies with immuno-evasive or immuno-suppressive characteristics. Nevertheless, translating this promising hypothesis into widespread clinical benefit will require sustained investigative rigor, iterative clinical validation, and a commitment to refining therapeutic protocols across diverse oncologic contexts.

The substantial risk of cytokine release syndrome with CAR T-cell treatment poses a significant impediment to targeting numerous immune checkpoint molecules simultaneously (173). However, safety measures that can slow the development of toxicity should be taken into account. One such measure is the use of suicide gene “safety switch” systems, such as iCaspase-9, in clinical studies (174). Overall, advances in this synergistic approach may lead to potentially curative therapies for patients with hematologic malignancies.

### Combination with targeted therapy

Multiple research studies are presently being conducted to ascertain the effectiveness and toxicity of combining targeted therapies with immunotherapies (mostly ICB) for cancer, as many targeted therapeutic agents have the ability to directly or indirectly alter immune cell function (117) (**Figure 2e**). Immunotherapies (mostly ICB) are currently being used in conjunction with nearly every targeted treatment that has been demonstrated to alter immune responses.

Since lymphoma cells’ surface antigens are the most accessible, monoclonal antibodies (mAb) that target these antigens have emerged as a key therapeutic approach for a variety of lymphoid cancers. Rituximab was the first monoclonal antibody to target CD 20 and the first to be authorized for use in cancer treatment. In the NCT03245021 trial, the efficacy of CD 20 in conjunction with nivolumab was examined (175). The overall remission rate (ORR) was 84% (16/19), of which 47% (9/19) achieved CR, 37% (7/19) achieved partial remission (PR), 5% (1/19) achieved disease stabilization, and 11% (2/19) achieved disease progression (PD) in best-practice remission (175). This implies that the combination of navulizumab and rituximab for follicular lymphoma treated as a first treatment has a favorable toxicity profile and high rates of total and partial remission, which may provide patients with an option to traditional chemotherapy. Vibrituximab (BV, ADCetris) is a CD 30 ADC that uses a valine-citrullinated peptide junction to connect an anti-CD 30 antibody to the anti-mitotic drug monomethyl auristatin E (MMAE). In patients with R/R PMBL, nabulizumab with BV demonstrated long-lasting safety and effectiveness in the NCT02581631 trial; no new safety signals were found (176).

The development of lymphoma is intimately associated with signal transduction pathways. Inhibitors targeting key pathways, including spleen tyrosine kinase (SYK), Bruton tyrosine kinase (BTK), phosphatidylinositol 3-kinase (PI3K)/AKT/mammalian target of rapamycin (mTOR), Janus kinase signaling and activator of transcription (JAK-STAT), NOTCH, NF-κB, and ubiquitin proteasomal pathway (UPP) have been used to treat lymphomas. For instance, PI3K inhibitors are authorized to treat lymphoma and breast cancer. Because PI3K-δ is expressed in immune cells, it has a direct impact on immune cells, changes local tumor metabolism, and downregulates antigen presentation mechanisms in addition to its direct anticancer effects (177). T-cell activity and quantity are known to be enhanced by the Bruton’s Tyrosine Kinase/Interleukin-2-inducible T-cell Kinase (BTK/ITK) inhibitor ibrutinib, which has been authorized for the treatment of non-Hodgkin lymphomas. By creating covalent connections with cysteine residues in the BTK active site, the irreversible BTK inhibitor ibrutinib reduces the activity of the BTK enzyme. With signaling pathway inhibitors, direct targeting of signaling pathways and off-target effects continue to be significant issues. An essential modulator of healthy myeloid and lymphoid development is the PI3K-AKT-mTOR pathway (178). When activated, the catalytic subunit of PI3K (p110α, p110β, p110γ, and p110δ) recruits AKT to the plasma membrane and begins PIP3 (179). AKT can activate mTOR, which is made up of two multiprotein complexes: mechanistic Target of Rapamycin Complex 1 (mTORC 1) and mTORC 2. To activate essential drivers of protein translation, mTORC 1 phosphorylates S6K1 and 4E-BP 1 (180). Mutations in PIK3CA and p110δ are the primary drivers of downstream B-cell receptor signaling. Growth factors that control cell survival, proliferation, differentiation, and apoptosis, as well as extracellular cytokines including interferon, IL-2, and IL-6, stimulate the JAK-STAT pathway (181). JAK 1/JAK 3 are immunomodulatory, whereas JAK 2 promotes erythrocyte and platelet production (182). JAK causes nuclear translocation, homodimerization, and STAT phosphorylation (183). STAT 1, STAT 2, STAT 3, STAT 4, STAT 5A, STAT 5B, and STAT 6 are the seven STAT proteins (182). One of the major signaling channels implicated in both neoplastic processes and healthy cellular functioning is the NF-κB pathway (184). The IκB kinase (IKK) complex, which inhibits NF-κB (IκB) proteins and NF-κB transcription factors, such as RelA/p65, RelB, c-Rel/Rel, p50, and p52, is the main element of the NF-κB pathway (185). B-cell-associated kinases (BAK), such as BTK or PI3K δ, are key signal transducers for BCR signaling that trigger cascade reactions to form the multiprotein CARD 11-BCL 10-MALT 1 (CBM) complex (186). The complex facilitates NF-κB activation by interacting with IKK, an NF-κB upstream molecule (187, 188). The majority of B-NHL types frequently exhibit constitutive activation of NF-κB (187).

DNA methylation, histone acetylation, and methylation are the primary components of epigenetic control. Chromatin state is controlled by histone acetylation and methylation. Although epigenetic changes are clinically significant, modulators that precisely target these changes have not yet been discovered. Many lymphoma subtypes have exhibited clinical effectiveness when treated with demethylating drugs and histone deacetylase (HDAC) inhibitors. The precise mode of action is yet unknown, though, and more research into biomarkers to forecast clinical success is required. Romidepsin and pembrolizumab together had an ORR of 50% in patients with r/r T-cell lymphoma, were durable in people over 60, and had a tolerable safety profile, according to the NCT03278782 trial (189).

The integration of ICIs with targeted therapies aims to leverage the complementary mechanisms of these two modalities. By combining immune modulation with targeted molecular intervention, this approach seeks to enhance anti-tumor efficacy while potentially reducing the risk of resistance. Some early studies explored the use of PD-1 inhibitors combined with HDACi in R/R cHL, demonstrating high response rates and deep remissions, offering a crucial alternative for patients unsuitable for chemotherapy. However, many other combination strategies encountered significant setbacks. Off-target effects of targeted drugs combined with the autoimmune-like toxicity of ICIs may produce additive or synergistic toxicity, leading to unexpected severe adverse events and narrowing the therapeutic window. However, although preclinical studies have supported the strategy of combining immune checkpoint blockade therapy with targeted therapy, further studies are needed to fully determine its safety and efficacy.

### Combination with oncolytic viruses

Strategies that use viruses to induce innate and adaptive immune responses and directly kill cancer cells have been extensively evaluated. Oncolytic viruses (OV) are natural or recombinant viruses that self-propagate, electively replicate in cancer cells, and infect neighboring cancer cells to induce a cytolytic effect. In particular, OVs are different from traditional cancer treatments because they have evolved naturally to effectively take over and rewire the host cellular machinery to produce therapeutic and viral transgenes at high levels (190). By infecting tumor cells to cause immunogenic cell death (which sets off an inflammatory reaction), OV has been demonstrated to activate the immune system (**Figure 2f**). This specific form of apoptosis causes the release of immunostimulants, which initiate innate and direct adaptive immune responses against cancer cells, by releasing pathogen-associated molecular patterns (PAMPS), tumor-associated antigens (TAAs), and danger-associated molecular patterns (DAMPS) from lysed tumor cells (191, 192). The APC in the TME then detects these critical compounds, triggering an immunological response. Furthermore, a systemic and enduring anti-cancer response is produced by this local immune system stimulation (193). In earlier research, recombinant engineered lyssaviruses that express monoclonal antibodies against the immunosuppressive chemical TIGIT were created. These recombinant lysosomal viruses have the ability to transform “cold” TME into “hot” and trigger a potent immune response against tumors (194). Furthermore, these viruses were more effective and promoted tumor regression when combined with PD-1 or LAG-3 inhibitors (195).

According to a number of preliminary publications, through the induction of anti-tumor M1-like polarization, the recruitment and function of T effector cells, the promotion of IFN-γ levels in the TME, and the downregulation of Treg density and activity, combination therapy using ICI and OV was observed to cause tumor regression (196, 197).

Although several additional viruses are in preclinical and clinical research, talimogene laherparepvec (T-VEC or Imlygic) is the only FDA-approved oncolytic virotherapy for metastatic melanoma at this time. Oncolytic viruses are believed to be specific for tumor cells and have a wide range of immune stimulation, in contrast to more conventional treatments like chemotherapy and radiation therapy, which lack tumor specificity for all replicating cells, and other immunotherapies, which are constrained in their application because they require the presence of particular ligands or receptors. The broad impact of lysosomal viruses is a result of the use of host-adapted immune responses that are able to acutely discriminate between target and non-target cells for precise specificity, as well as the ability to take advantage of signals that may be prevalent for all malignancies199.

Currently, several trials are investigating the possibility of lysosomal viruses as monotherapy or in combination with immune checkpoint therapy against PD-1. However, challenges such as viral delivery efficiency, immune-mediated viral clearance, and management of dual toxicity profiles require further optimization. Ongoing research aims to engineer next-generation oncolytic viruses with improved tumor tropism and immune-modulating transgenes, while exploring biomarker-driven strategies to maximize therapeutic synergy in lymphoma patients.

### Combination with antiangiogenic agent

Abnormal angiogenesis is a hallmark of cancer (198). Cancer is characterized by abnormal angiogenesis (199), which also favorably regulates TME (200) (**Figure 2f**). MDSC accumulation and tumor-associated macrophage (TAM) differentiation into immunosuppressive M2 macrophages are accelerated by hypoxia (201). Additionally, by upregulating CC chemokine ligands, hypoxia indirectly promotes the degradation of TCR. Additionally, it suppressed immune cell activation by upregulating PD-L1 expression in cancer cells, TIM-3 and CTLA-4 expression in TAM, MDSC, and TCR, and indirectly upregulating PD-1 expression in CD8+ T cells. Immune effector cells cannot penetrate the cancer lesion due to increased tumor interstitial fluid pressure (TIFP), which is caused by decreased lymphatic channels and increased tumor vascular permeability (202). Antiangiogenic medicines reduce the activity of immunosuppressive cells (e.g., MDSC and TME) and normalize immature blood vessels, hence reprogramming the TME (203). In patients with advanced melanoma receiving PD-1-targeted immunotherapy, we have shown that intra-tumoral hypoxia is linked to reduced TIL function, PFS, remission duration, and a shorter overall survival (204). Vascular Endothelial Growth Factor Receptor (VEGFR)-specific tyrosine kinase inhibitors (TKIs), which are angiogenesis inhibitors, may also favorably regulate TME (205), boost the infiltration of mature dendritic cells and neutrophils, and decrease MDSC, Treg cells, and macrophages (206). Consequently, it is postulated that by using complimentary modes of action, VEGFR inhibitors and immune checkpoint inhibitors together may enhance therapeutic effect. Clinical trials of PD 1-targeted or PDL 1-targeted immune checkpoint inhibitors in combination with VEGF-targeted TKIs have been reported or are in progress, including in the first-line or PD 1-refractory setting. This combination has demonstrated promise in a number of preclinical models in various tumor types.

In fact, anti-angiogenic treatments have only been created to fight cancer by decreasing the vascular network and preventing the formation of new blood vessels, which stops the tumor cells from receiving oxygen and nutrients. Targeting VEGF function is a major component of authorized angiogenesis inhibitors for tumor therapy, given the pivotal role of VEGF signaling in angiogenesis. These substances not only alter angiogenesis but also improve immunotherapy because of VEGF immunomodulatory action (207). Therefore, by improving immune cell recruitment and induction, angiogenesis inhibitors transform TME from immunosuppressive to immunosupportive. The FDA has approved several powerful angiogenesis inhibitors to date, including axitinib, bevacizumab, cabozantinib, everolimus, lenalidomide, levatinib mesylate, pazopanib, ramorubicin, regorafenib, sorafenib, sunitinib, thalidomide, vandetanib, and Ziv-abacip (208). The first FDA-approved VEGF-targeting medication, bevacizumab, can be used alone or in combination with other therapeutic agents to treat a range of human tumors, including breast, ovarian, cervical, NSCLC, RCC, and colorectal cancers (209).

## Immune-related adverse events

Immune-related adverse events (irAE) represent a distinct class of side effects arising from immune system hyperactivation during treatment with immune checkpoint inhibitors (ICIs), such as anti-PD-1/PD-L1 and anti-CTLA-4 agents. These events occur when T cells, released from immunosuppressive constraints, aberrantly target healthy tissues. While conventional anticancer therapies often face limitations due to drug resistance, combining ICIs with other modalities—including targeted therapy, antiangiogenic agents, radiotherapy, chemotherapy, and surgery—has emerged as a promising strategy to overcome resistance and enhance therapeutic efficacy. Such combinations may amplify anticancer effects by improving antigen presentation, replenishing exhausted effector T cells, and stimulating immune-mediated tumor cell destruction through antigen release (210). However, the immunological rewiring and tumor cell death pathways modulated by these therapies can also influence immunotherapy outcomes.

A critical challenge in combination regimens is the increased incidence of severe adverse events (AEs), particularly irAE, which are categorized into three groups: common AEs (e.g., fatigue, diarrhea, rash), organ-specific AEs (e.g., colitis, hepatitis, pneumonitis), and systemic inflammatory conditions. Although most irAE are mild to moderate, severe or life-threatening manifestations—such as cardiac and neurologic toxicities—demand urgent intervention (211). Unlike chemotherapy-induced toxicities, irAE exhibit delayed onset, prolonged duration, and unique risk profiles, necessitating tailored management strategies (212). Additional immunosuppressive medications may occasionally be required, even though glucocorticoids are frequently used to improve mild and severe irAE (213, 214). Notably, while combination therapies elevate AE frequency, most events remain manageable through prompt drug discontinuation and corticosteroid administration (154, 215). Documented protocols emphasize that early irAE detection and intervention—including drug withdrawal and immunosuppression—can prevent progression and reverse toxicity in many cases (216).

In summary, irAE are characterized by heterogeneous presentations, insidious onset, and nonspecific symptoms. To optimize patient outcomes, clinicians must prioritize comprehensive management strategies encompassing five key domains: prevention, risk assessment, proactive screening, timely therapy, and continuous monitoring (217).

## Precision biomarkers for efficacy

Currently, immunological checkpoints in conjunction with other medicines have shown promise in the treatment of lymphomas, and a range of biomarkers are crucial in predicting efficacy and assessing prognosis.

Numerous cytokines and tumor cell exosomes within the tumor microenvironment can induce PD-L1 expression, promoting tumor immune escape. The TME primarily comprises the vascular system, extracellular matrix, non-malignant cells surrounding the tumor, and a complex network of signaling molecules that maintain connections within the microenvironment. This composition facilitates the growth, proliferation, invasion, and metastasis of malignant cells. Extracellular exosomes carrying non-coding RNA represent another component within the TME that promotes tumor cell proliferation and evolution (218). First, the presence of tumor-infiltrating T cells has been demonstrated to correlate with clinical benefit from anti-PD-1/PD-L1 therapy. Second, immunosuppressive cell populations within the tumor microenvironment may inhibit responses to PD-1/PD-L1 blockade. Third, molecular characteristics of the tumor microenvironment before and after anti-PD-1/PD-L1 immunotherapy can serve as alternative response biomarkers (170).

In current clinical practice, PD-L1 detection primarily relies on immunohistochemistry (IHC). Kiyasu et al. (219) demonstrated through a large-scale series of 1,200 DLBCL samples that PD-L1-positive DLBCL patients exhibit lower overall survival rates than PD-L1-negative DLBCL patients. Furthermore, patients with PD-L1-positive tumor cells accompanied by low PD-1-positive TIL counts exhibited poorer prognosis compared to those with PD-L1-negative DLBCL and high PD-1-positive TIL counts (219). Regarding the prognostic impact of PD-1/PD-L1 expression in FL, conflicting data have been reported. Carreras et al. demonstrated an association between high PD-1+ cell counts and improved overall survival (OS) and progression-free survival (PFS), although these findings remain controversial (220). Compared to PD-1, the prognostic relevance of PD-L1 staining in FL has been studied less extensively to date. Only one study reported a significant correlation between tumor cell PD-L1 expression and reduced survival in FL patients (221). Preliminary data from HL clinical trials suggest that patients with PD-L1+ tumor cells derive the greatest benefit from PD-1/PD-L1 immune checkpoint blockade therapy (222). However, substantial responses have also been observed in some PD-L1- patients (223). This observation highlights the limitations of using PD-L1 protein expression alone as a sole predictive biomarker, as its expression is heterogeneous within tumor cells and can increase spontaneously or following treatment (222).

In solid tumors and cHL, higher PD-L1 expression in tumor cells as determined by immunohistochemistry has been linked to improved anti-PD-1 therapeutic response (224, 225). PD-L1 immunohistochemistry antibodies, however, have not been standardized and come in a variety of clones. Likewise, soluble PD-L1 blood levels as determined by the enzyme-linked immunosorbent assay (ELISA) could be a prognostic biomarker for PCM or DLBCL patients (226). However, since these patients receive traditional chemotherapy, research that focuses on the PD-1 pathway needs to be done. TILs, especially those that express PD-L1, are linked to a greater response to PD-1-targeted treatment in patients with solid (227). However, little information exists on PD-L1-expressing TILs in lymphoma patients. Response can be predicted by using immunohistochemistry to evaluate the kinetics of immune cell profiles in biopsy sample TME at various stages of the treatment regimen.

Tumor mutation burden (TMB) is defined as the total number of substitutions per megabase in the coding regions of target genes (including synonymous mutations), representing the sum of somatic mutations in the tumor genome after removing germline mutations. The CheckMate 026 study demonstrated that in patients with high TMB (≥243 missense mutations), treatment with the PD-1 inhibitor nivolumab significantly improved progression-free survival (PFS) and overall survival (OS) compared to conventional chemotherapy (228). TMB also predicts PD-L1 inhibitor efficacy. Although TMB is considered a strong predictor for immunotherapy, its predictive value may be conditional. Cases exist where patients with high TMB exhibit immune non-response, while those with low TMB demonstrate favorable immune responses. Clarifying the conditions under which TMB exerts its predictive role is therefore critical.

To forecast the immunogenicity of cancer germline antigens or mutagen-derived neoantigens, as well as their binding affinity to immune cells, a computational genomic method has been developed. Additionally, it can forecast how well anti-PD-1 or anti-CTLA-4 medications will work (229, 230). Although the feasibility of computational genomic methods for solid tumors has been demonstrated, the paucity of data raises doubts about the suitability of mutational burden as a biomarker for lymphoma (231). Since the majority of the data comes from patients who have solid tumors or models of solid tumors, not all of the aforementioned methods apply to hematologic malignancies.

In the future, with advances in genomics, transcriptomics, and immunoassay technologies, the use of combination therapies involving multiple immune checkpoint inhibitors represents a new developmental trend. Establishing comprehensive biomarker evaluation systems through novel approaches such as computational biology and bioinformatics can enable more accurate prediction of immune checkpoint inhibitor efficacy, thereby advancing the development of precision oncology.

## Conclusions and outlook

With the in-depth study of the immune microenvironment of lymphoma, immune checkpoint inhibitors in combination with other therapies show a broad application prospect in lymphoma treatment. Immune checkpoint inhibitors enhance the anti-tumor activity of T cells by blocking the immunosuppressive signaling pathway, while the combination with other therapies further enhances the therapeutic effect. This review elucidates the mechanistic rationale and clinical significance of combining immune checkpoint inhibitors with conventional therapies (chemotherapy, radiotherapy), targeted agents, and emerging treatment modalities. The combination of immune checkpoint inhibitors with other therapeutic means has also made positive progress. For example, when used in combination with chemotherapy, radiotherapy, targeted therapy, CAR-T cell therapy, etc., they are able to enhance anti-tumor immune responses at multiple levels. However, combination therapy also faces some challenges, such as the increased incidence of immune-related adverse events and the optimization of treatment sequence and dosage. Meanwhile, the varied efficacy of these combination therapies highlights the necessity for a refined predictive framework to anticipate treatment outcomes and guide personalized treatment plans.

With the advancement of new technologies and deepening research, tumor immunotherapy approaches continue to diversify and evolve. However, numerous challenges persist, such as only a small proportion of patients achieving complete and durable immune responses, imperfect prediction and management of immune-related adverse events, and the lack of effective and reliable biomarkers for predicting treatment efficacy. Realizing precision personalized immunotherapy remains fraught with difficulties. The journey toward precision oncology continues. The shift from universal treatment models to precision medicine represents an irreversible future trend. Immunotherapy has ushered in a new therapeutic revolution, emerging as a pivotal cancer treatment modality. The approval of the first tissue-agnostic indication for anti-tumor therapies defined by biomarkers—notably microsatellite instability-high/mismatch repair deficiency (MSI-H/dMMR)—marks the commencement of a new era in precision oncology. Clinical studies have demonstrated that patients with various MSI-H tumors can achieve significant survival improvements through immunotherapy. Rapid advancements in biotechnology, particularly in gene sequencing, have paved the way for precision oncology. Future developments will likely yield novel, safer precision treatment strategies based on human genetics and genomics, next-generation sequencing, signaling pathways, gene interactions and networks, molecular regulation and control, or functional mechanisms. Advancements in biomedical technologies have deepened clinical understanding of cancer, while the significant reduction in genomic sequencing costs has accelerated the establishment and expansion of precision oncology databases. These developments provide essential foundations for refining and advancing new precision treatment paradigms. Building on this foundation, clinicians will develop optimized drug combinations and improved delivery methods to enhance efficacy while reducing adverse reactions. The goal is to strive for longer survival times and better quality of life for cancer patients. This requires mandatory implementation of biopsies and liquid biopsies, combined with “basket trial” designs to achieve precise patient stratification. Prospective studies are urgently needed to directly compare different administration strategies (e.g., immune induction, concurrent combination, consolidation maintenance) to determine the optimal sequence within each combination regimen. This approach will maximize synergistic effects while minimizing overlapping toxicities. Establish a large-scale international patient registry to collect real-world efficacy data on combination therapies in clinical practice. This evidence is crucial for validating randomized trial results, understanding long-term outcomes, identifying rare toxicities, and optimizing treatment strategies for heterogeneous patient populations.

In the future, with further research on the immune microenvironment and biomarkers, it is expected to develop more precise combination therapy regimens to improve the survival rate and quality of life of lymphoma patients. Overall, the integration of immune checkpoint inhibitors with complementary therapies holds transformative potential, yet requires rigorous mechanistic exploration and clinical validation to maximize the therapeutic index and durability of responses. Meanwhile, in-depth exploration of the mechanism and optimization of combination therapy will provide a new direction for immunotherapy of lymphoma.
